# Election of Officers

**Published:** 1873-11

**Authors:** 


ELECTION OF OFFICERS. At the annual meeting of the Chautauqua County Medical Society, the following officers were elected:

*President*, Dr. Ai Waterhouse, Jamestown. *Vice President*, Dr. S. M. Smith, Dunkirk. *Sec'y and Treas'r*, Dr T Chas. Wilson, Portland.

The Semi-Annual meeting will be held at Jamestown, on the second Tuesday of January, 1874.

